# Does statin use have a disease modifying effect in symptomatic knee osteoarthritis? Study protocol for a randomised controlled trial

**DOI:** 10.1186/s13063-015-1122-2

**Published:** 2015-12-23

**Authors:** Yuanyuan Wang, Andrew Tonkin, Graeme Jones, Catherine Hill, Changhai Ding, Anita E. Wluka, Andrew Forbes, Flavia M. Cicuttini

**Affiliations:** Department of Epidemiology and Preventive Medicine, Monash University, Alfred Hospital, Melbourne, 3004 Australia; Menzies Institute for Medical Research, University of Tasmania, Private Bag 23, Hobart, TAS 7000 Australia; The Queen Elizabeth Hospital, University of Adelaide, Woodville, SA 5011 Australia; Discipline of Medicine, University of Adelaide, Adelaide, SA 5005 Australia

**Keywords:** Statins, Osteoarthritis, Cartilage, Pain, Magnetic resonance imaging

## Abstract

**Background:**

Osteoarthritis (OA) is a major clinical and public health problem, with no current medications approved as having disease modifying effects. HMG-CoA reductase inhibitors, or “statins”, a drug class widely used to prevent cardiovascular events, could potentially affect OA progression via a number of mechanisms including their effects on lipid metabolism and inflammation. The aim of this multicentre, randomised, double-blind, placebo-controlled trial is to determine whether atorvastatin reduces the progression of knee structural changes and symptoms over 2 years in patients with symptomatic knee OA.

**Methods/design:**

350 patients with symptomatic knee OA will be recruited through the OA Clinical Trial Network (in Melbourne, Hobart and Adelaide). They will be randomly allocated to the two arms of the study, receiving either 40 mg of atorvastatin or identical placebo once daily for 2 years. Magnetic resonance imaging of the knee will be performed at baseline and 2 years later. Knee structure, symptoms and function will be assessed using validated methods. The primary outcome is annual percentage change in knee cartilage volume. Secondary outcomes include progression of cartilage defects, bone marrow lesions, knee pain and function. The primary analysis will be by intention to treat, but per protocol analyses will also be performed.

**Discussion:**

The study will provide high-quality evidence to address whether atorvastatin has a novel disease modifying effect in OA by delaying the structural and symptomatic progression of knee OA. Thus, the trial has major public health and clinical importance, as if found to be beneficial, atorvastatin could produce substantial cost savings by delaying and possibly reducing the need for joint replacement surgery, and provide marked improvements in quality of life for people with OA.

**Trial registration:**

Australian New Zealand Clinical Trials Registry: ACTRN12613000190707, registered on 18 February 2013.

## Background

Osteoarthritis (OA), characterised by progressive structural deterioration and symptoms, is a significant clinical and public health problem, causing pain, disability and a substantial health care burden. It is a major contributor to the burden of disease, being ranked as the eleventh highest contributor of global disability among 291 conditions in the Global Burden of Disease study and associated with 71.1 million years lived with disability, an increase of 64 % from 1990 to 2010 [1]. Symptomatic knee OA makes a significant impact on society, with 44.7 % of people developing this condition in their lifetime [2]. Current treatments for OA mainly focus on the improvement of symptoms. Although several drugs and nutraceuticals have been examined for their effects on slowing structural progression of OA over the past 10 years, none has been approved as a disease modifying OA drug [3]. Many patients with symptomatic end-stage OA are eventually faced with joint replacement as the only treatment option to improve quality of life, and the number of joint replacements has continued to increase worldwide.

There is growing evidence that OA is a multifactorial complex disorder, with a significant metabolic component in its pathogenesis, in which various interrelated lipid, metabolic and inflammatory mediators contribute to the initiation and progression of the disease [4–6]. These metabolic factors appear to be more important in OA of the knee than the hip [7].

In the Chingford study of 1,003 middle-aged women, hypercholesterolaemia was associated with increased risk of knee OA independent of obesity, and the association was stronger for bilateral knee OA than for unilateral knee OA [8]. In the Ulm study of 809 patients with joint replacement due to OA, high serum cholesterol levels were independently associated with increased risk of knee OA [9]. In recent longitudinal magnetic resonance imaging (MRI) studies, we showed that higher serum cholesterol and triglyceride levels were associated with adverse structural changes (increased incidence of bone marrow lesions, BMLs) in asymptomatic middle-aged women over 2 years [10], while higher serum levels of high-density lipoprotein (HDL) cholesterol were protective against worsening of BMLs in older adults over 2.7 years [11]. BMLs are early knee structural changes associated with knee pain and cartilage loss [12, 13] and joint replacement in OA [14].

Inflammation has also been implicated in the pathogenesis of OA [6]. In human OA, the histological changes include increased vascularity, lining layer thickening, and inflammatory cell infiltration with increased numbers of lining cells. A mixed population of inflammatory cells (macrophages, activated T cells) is seen in the sublining tissue [15]. These cells, as well as chondrocytes, produce pro-inflammatory cytokines, such as interleukin (IL)-1β, IL-6, and tumour necrosis factor (TNF)-α [15], which are detectable even in early OA [15]. Synovitis identified by arthroscopy predicted increased cartilage loss over 1 year [16], and synovitis detected by contrast-enhanced MRI was associated with increased cartilage loss over 30 months [17]. Circulating levels of C-reactive protein were associated with disease progression and decreased cartilage volume at the knee [18, 19]. Increased serum level of IL-6 was a significant predictor of radiographic OA 5–10 years later in women [20], associated with increased knee cartilage loss over 2 years in OA [21] and increased cartilage volume loss over 3 years in older adults [22]. We found that elevated TNF-α levels were also associated with increased rate of cartilage volume loss over 3 years in older adults [22].

Hydroxy-methyl-glutaryl coenzyme A (HMG-CoA) reductase inhibitors (statins), a drug class used to lower low-density lipoprotein (LDL) cholesterol levels with smaller effects on lowering triglycerides and increasing HDL cholesterol levels, are proven and have been widely used to prevent cardiovascular events [23]. In addition to lowering LDL cholesterol, statins have a broad range of pleiotropic effects, including anti-inflammatory effects, which could exert an effect on synovium and cartilage [24]. Data from observational studies of statins are somewhat conflicting (Table 1). Some large cohort studies have shown that statin use is associated with reduced incidence and progression of OA [25, 26] and that higher statin dose and larger statin increments are associated with reduced incident episode of clinically defined OA [27]. However, other cohort studies have reported that statin use is associated with an increased incidence of OA and arthropathy [28, 29], or that statin use is not associated with improvement in knee pain, function, or structural progression of knee OA [30]. Thus, the current evidence for the association between statin use and the risk of OA comes from observational human studies which are subject to bias and confounding, and the overall data are inconclusive. No randomised controlled trials of statins have been performed so far.

**Table 1:** Summary of findings from human epidemiological studies for association between statin use and risk of osteoarthritis

Author, year	Study design	Participants	Outcome measure	Main results
Beattie MS, et al. 2005 [29]	Prospective cohort study	5,674 elderly women aged ≥65 years from the Study of Osteoporotic Fractures	Radiographic hip OA	Statin use was associated with an increased risk of developing radiographic hip OA, but did not adversely affect the progression of established disease.
Chodick G, et al. 2010 [26]	Retrospective population-based cohort study	193,770 individuals from the computerised medical databases of a large health organisation	International Classification of Diseases, 9th revision, diagnosis codes	Persistent statin use was associated with a reduced incidence of OA.
Clockaerts S, et al. 2012 [25]	Prospective population-based cohort study	2,921 participants aged ≥55 years in the Rotterdam study	Radiographic knee and hip OA	Statin use was associated with reduced incidence and progression of radiographic knee OA, but not radiographic hip OA over 6.5 years.
Kadam UT, et al. 2013 [27]	Retrospective cohort study	16,609 adults aged ≥40 years from the UK General Practice Research Database	Clinically defined OA: OA-related diagnostic categories from a standard clinical classification, recorded by GPs in the actual consultations	Higher statin dose and larger statin increments were associated with reduced incident episode of clinically defined OA over 2–10 years.
Mansi IA, et al. 2013 [28]	Retrospective cohort study	92,360 patients in the San Antonio Military Multi-Service Market	International Classification of Diseases, 9th edition, diagnosis codes	Statin use was associated with an increased incidence of OA and arthropathy over 4 years.
Riddle DL, et al. 2013 [30]	Prospective cohort study	2,207 participants with radiographically suspected or confirmed knee OA in the Osteoarthritis Initiative	Knee pain, function, and radiographic knee OA	Statin use was not associated with improvement in knee pain, function, or structural progression of knee OA over 4 years.

We will conduct a randomised controlled trial to determine whether atorvastatin has a disease modifying effect in OA by reducing the MRI-assessed structural progression (knee cartilage volume loss, progression of cartilage defects and BMLs) and symptoms in patients with symptomatic knee OA over 2 years. It was hypothesised that atorvastatin use will reduce the rate of knee cartilage volume loss (primary hypothesis), the progression of cartilage defects and BMLs (secondary hypothesis), and improve symptoms and function (secondary hypothesis) over 2 years compared with placebo in people with symptomatic knee OA. If statins are proven to be effective, they will offer a novel therapeutic approach to reducing the progression of knee OA.

## Methods/design

### Study design

The Osteoarthritis of the Knee Statin (OAKS) study is a multicentre, randomised, double-blind, placebo-controlled trial over 2 years. The trial was registered at the Australian New Zealand Clinical Trials Registry prior to recruitment, and trial reporting will be guided by the CONSORT Statement [31]. A total of 350 patients with symptomatic knee OA will be recruited in equal numbers via the OA Clinical Trial Network in Melbourne, Hobart and Adelaide, using a combined strategy including collaboration with general practitioners, rheumatologists, and orthopaedic surgeons, as well as advertising through local media. Ethics approval has been obtained from The Alfred Hospital Ethics Committee (521/12), Monash University Human Research Ethics Committee (CF13/595 - 2013000236), Tasmania Health and Medical Human Research Ethics Committee (H0012971), and Human Research Ethics Committee (TQEH/LMH/MH) (HREC/13/TQEHLMH/20). Written informed consent will be obtained from all participants.

### Inclusion criteria

The inclusion criteria will be: males and females with symptomatic knee OA for at least 6 months with a pain score of at least 20 mm on a 100-mm visual analogue scale (VAS); age 40–70 years old; and meeting the American College of Rheumatology (ACR) criteria for symptomatic knee OA [32], assessed by a rheumatologist.

### Exclusion criteria

Participants with any of the following conditions will be excluded: inability to give informed consent; severe radiographic knee OA (grade 3 according to Altman’s atlas [33]) or severe knee pain (on standing >80 mm on a 100-mm VAS); rheumatoid arthritis or other inflammatory arthritis; significant knee injury; familial hypercholesterolaemia for which statins are indicated, known atherosclerotic cardiovascular disease, diabetes mellitus, taking current lipid lowering therapy, or with previous adverse reaction to statins; absolute cardiovascular risk estimated using the Framingham Risk Equation of more than 15 % within the next 5 years (National Heart Foundation of Australia, 2005); fasting total cholesterol level >7.5 mmol/L; clinically significant renal disease or abnormal liver function assessed by aspartate aminotransferase and alanine aminotransferase, creatine kinase more than twice the upper limit of laboratory normal range; patients undergoing arthroscopy or open surgery in the index knee in the last 12 months; receiving intra-articular therapy in the index knee in the last 12 months; concomitant use of potent analgesics including opiates; co-morbidity that may limit participation (such as planned index knee joint replacement or medical conditions, for example, malignancy in the past 5 years other than non-melanoma skin cancer); relocation; any contraindication to MRI scanning (for example, implanted pacemaker, metal sutures, presence of shrapnel or iron filings in the eye, or claustrophobia); pregnancy, breast feeding, or women trying to become pregnant.

### Randomisation and blinding

Allocation of participants in a 1:1 ratio to either the intervention or control group will be based on computer-generated random numbers prepared by a statistician with no involvement in the trial. Block randomisation will be performed using a central automated allocation procedure, stratified according to the study site. The randomised controlled trial will be a double-blind one, with both participants and investigators assessing outcomes blinded to treatment allocation. Allocation concealment and double blinding will be ensured by 1) the medications being dispensed by the hospital clinical trial pharmacy in each site; 2) the use of an identical placebo tablet; 3) objective measures of knee structural changes being made by trained observers blinded to group allocation and the time sequence of MRI scans; 4) subjective measures being taken by research assistants blinded to group allocation.

### Intervention

All participants will be provided usual care by their treating health practitioners. Participants in the intervention arm will receive 40 mg of atorvastatin (Sandoz) once daily, and those in the control arm will receive 40 mg of inactive matching placebo (Pharmaceutical Packaging Professionals Pty Ltd, Thebarton, South Australia) once daily. Atorvastatin was chosen as it is the most widely prescribed statin and has potent LDL cholesterol lowering effects.

### Study procedure

At screening, participants will complete questionnaires, have a knee X-ray, and undergo biochemical testing including liver function tests, creatine kinase and renal function tests, to ensure inclusion criteria are met. The study knee will be defined as the one with symptomatic OA; if both are symptomatic, the one with least severe radiographic OA (joint space narrowing) will be studied, to reduce loss to follow-up for joint replacement. After screening, study visits will be scheduled for 0 (baseline), 6, 12 and 24 months (Table 2). The same researchers, who are blinded to treatment allocation, will measure all clinical variables, administer questionnaires, monitor compliance, and record adverse events at these visits. Compliance by trial medication will be assessed by pill count. Biochemical testing will be performed at 4 weeks, and at 6, 12 and 24 months. At baseline and 24 months the index knee will be imaged on the same whole-body MRI unit.

**Table 2:** Timetable and measures to be made

	Screening	Month 0 (baseline)	Week 4	Month 6	Month 12	Month 18	Month 24
X-ray	√						
Biochemical testing	√		√	√	√		√
Questionnaires	√	√		√	√		√
Knee VAS	√	√		√	√		√
Knee WOMAC		√		√	√		√
IPAQ		√					√
SF-36		√					√
Concomitant medications	√	√		√	√		√
Smoking		√					√
History of joint disease	√	√					√
Co-morbidities	√	√					√
Education and occupation		√					
Physical examination		√					√
Height and weight		√					√
Lower limb muscle strength		√			√		√
Knee MRI		√					√
Compliance and adverse events				√	√	√	√
Medication dispensing		√		√	√	√	

### Quality assurance

To ensure high-quality execution of the trial in accordance with the protocol, all trial staff will be trained by the chief investigators and provided a standard protocol book (with details of standard operating procedures used for all trial contacts, visits, measurements, and monitoring) and case report forms.

### Outcome measures

#### Knee MRI

Knee MRI acquisition at the three study sites is presented in Table 3, including details of sequences and parameters being used.

**Table 3:** Magnetic resonance imaging sequences and parameters at three study sites

	Machine and coil	T1 sagittal	Proton density sagittal
Melbourne	3.0 T whole body MR unit (Achieva, Philips Medical Systems), using a commercial 16-channel transmit-receive knee coil	T1-weighted fat suppressed 3D gradient recall acquisition in the steady state; flip angle 15 degrees; repetition time 25.9 msec; echo time 9.2 msec; field of view 16 cm; 320 × 320 matrix; one acquisition; partition thickness 0.5 mm	Proton density fat-saturated acquisition; flip angle 90 degrees; repetition time 3,817 msec; echo time 25 msec; field of view 16 cm; 720 × 720 matrix; slice thickness 2.5 mm
Hobart	1.5 T whole-body MR unit (GE-Signa; GE Healthcare, Buckinghamshire, UK) using a dedicated 8-channel knee coil	T1-weighted fat-saturated 3D gradient-recalled acquisition; flip angle 30 degrees; repetition time 31 msec; echo time 6.8 msec; field of view 16 cm; 512 × 512 matrix; one excitation; slice thickness 1.5 mm	Proton density fat-saturated 2D fast spin echo sequence; flip angle 150 degrees; repetition time 3,800 msec; echo time 39 msec; field of view 16 cm; 512 × 512 matrix; 3 excitations; slice thickness 3 mm
Adelaide	1.5 T whole-body MR unit (Aera, Siemens) using a dedicated 15-channel transmit-receive knee coil	T1-weighted fat-saturated 3D gradient-recalled acquisition; flip angle 30 degrees, repetition time 14.7 msec; echo time 6.74 msec; field of view 16 cm; 448 × 448 matrix; one excitation; slice thickness 1.5 mm	Proton density fat-saturated 2D fast spin echo sequence; flip angle 180 degrees; repetition time 3,200 msec; echo time 39 msec; field of view 16 cm; 320 × 320 matrix; 1 excitation; slice thickness 3 mm

#### Primary outcome measure: annual percentage change in tibial cartilage volume

From sagittal T1-weighted images, medial and lateral tibial cartilage volume will be isolated by manually drawing disarticulation contours around the cartilage boundaries on a section-by-section basis. Measurement error and bias will be reduced by ensuring that one reader measures participants’ paired set of images blinded to time sequence, with a second reader performing independent consistency checks. In our previous study, we demonstrated a coefficient of variation of 2.0–3.4 % for this method [34]. We and others have shown that change in tibial cartilage volume over 2 years can be measured using this method and is both statistically and clinically significant in patients with symptomatic knee OA [34–36]. The annual percentage change in tibial cartilage volume will be calculated as (follow-up cartilage volume – baseline cartilage volume)/baseline cartilage volume/time between MRI scans*100.

#### Secondary outcome measures

##### Progression of cartilage defects

Cartilage defects will be graded at tibial and femoral sites (0–4) from sagittal images [37]. If multiple cartilage defects are present at one site, the highest grade will be recorded. Our intra- and inter-observer reliability for this method is 0.89–0.94 and 0.85–0.93, respectively [37]. The progression of cartilage defects will be defined as any increase in cartilage defect score in either tibial or femoral cartilage over 2 years.

##### Progression of BMLs

BMLs will be defined as discrete subchondral areas with increased signal intensity and ill-defined margins on sagittal proton density images, and graded at tibial and femoral sites (0–3). If multiple lesions are present at one site, the highest grade will be recorded. The intra- and inter-reader correlation coefficient for this method has been reported to be 0.88–0.93 [13]. The progression of BMLs will be defined as any increase in their grade in either tibial or femoral site over 2 years. The maximum areal size will also be measured for each lesion. The lesion with the largest size will be used if more than one lesion is present at the same site. Our intra-observer correlation coefficient for this method is 0.97 [38]. The change in areal size of BMLs over 2 years will be assessed since it is more sensitive to change, with positive results seen over 6 months in a previous clinical trial [39].

##### Change in knee symptoms and function

Knee pain, stiffness and function will be assessed using a self-administered validated questionnaire, WOMAC (the Western Ontario and McMaster Universities Osteoarthritis Index) [40], at each study visit. It will be used to describe the population, and change in symptoms and function measured by change in WOMAC score will be a secondary outcome.

### Other measurements

#### Anthropometry

Height (stadiometer), weight (electric scale) and body mass index (height/weight^2^) will be measured at baseline and 2 years.

#### Radiographic knee OA

A weight-bearing anteroposterior radiograph of the study knee will be scored for osteophytes and joint space narrowing on a four-point scale (0–3) using the Altman atlas [33]. Our intra- and inter-observer reliability is 0.93 and 0.86 for osteophytes and 0.93 and 0.85 for joint space narrowing, respectively [41].

#### Knee angle

The knee angle will be measured from weight-bearing radiographs, as it affects cartilage volume change [42]. Our intra-observer correlation coefficient is 0.98 [42].

#### Tibial plateau bone area

The cross-sectional area of tibial plateau will be measured manually from the reformatted axial MR images. The coefficient of variation of this method is 2.3–2.4 % [34]. Cartilage volume will be adjusted for bone area to account for joint size.

#### Meniscal tear and extrusion

The presence of meniscal tears and extent of meniscal extrusion will be determined from sagittal images and confirmed in coronal and axial images [43], as they affect cartilage volume change [43]. The intra- and inter-reader correlation coefficient has been reported to be 0.85–0.96 [43].

#### Physical activity

Physical activity will be measured using the International Physical Activity Questionnaire (IPAQ) [44] short version at baseline and 2 years.

#### Lower limb muscle strength

Lower limb muscle strength is expected to increase with a decrease in pain, and therefore will be measured by dynamometry at months 0, 12 and 24. The muscles measured in this technique are mainly quadriceps and hip flexors. The devices will be calibrated by suspending known weights at regular intervals. Repeatability estimates (Cronbach’s) were 0.91 [45].

#### Concomitant medication use

Concomitant medication use including glucosamine, chondroitin, corticosteroids and non-steroidal anti-inflammatory drugs will be allowed during the trial but will be documented. There is no definitive data showing that these treatments affect cartilage loss. The randomisation process is the most effective method for ensuring that two groups are as similar as possible with respect to known confounders and unknown potential confounders including treatments. We will adjust for medication use in the analyses.

#### Cigarette smoking history

Cigarette smoking history will be assessed at baseline and 2 years. As it affects cartilage loss and BMLs [46], we will adjust for smoking status in the analyses.

#### Knee injury

Knee injury will be assessed at baseline and 2 years. Prior knee injury is an exclusion criterion. Any significant injury during the study will be documented, as it is a risk factor for knee OA.

### Safety assessment

Adverse events will be monitored throughout the study. Standard safety and efficacy monitoring will be performed through regular face-to-face visits and phone calls between visits. The participants are requested to report any adverse events to the research staff spontaneously. Biochemical testing (liver function tests, creatine kinase and renal function tests) will be performed at 4 weeks, and at 6, 12 and 24 months. Details of the adverse event and its relationship with study intervention will be recorded and reported to the Ethics Committees.

### Sample size calculation

#### Primary outcome

Based on our previous published data, the rate of tibial cartilage volume loss in the control group was 3.0 +/− 3.0 per annum [47]. A 33 % reduction (that is, 1 %) in this rate is clinically significant since this indicates a significant reduction in structural progression of cartilage damage. Our previous work has shown that a 1 % reduction in the annual rate of cartilage volume loss over 2 years decreased the need for knee replacement surgery by 20 % over a 3-year period based on the strong association between the rate of cartilage volume loss and subsequent knee replacement [48]. With 140 participants in each arm, we will have 80 % power to detect a 30 % reduction in the rate of cartilage volume loss in the intervention group (2.0 %) compared with the control group (3.0 %), with alpha error 0.05, two-sided significance. Given our previous experience in such studies, we expect a maximum dropout rate of 20 % over 2 years. Therefore, a total of 350 participants (175 in each arm of the study) will be recruited.

#### Secondary outcome

Based on our reported progression rate of 68 % for cartilage defects [49] and 21 % [14] for BMLs over 2 years, with 140 participants per arm we will have 80 % power to detect a 17 % difference in the progression of cartilage defects and a 12 % difference in the progression of BMLs between the intervention group and the control group. With 140 in each arm, we will be able to detect a 30 % reduction in the WOMAC pain subscale in the intervention group.

### Statistical analysis

The primary analyses will be intention-to-treat analyses of primary and secondary outcomes. Per protocol analyses (according to protocol adherence for patients who have taken all the study medication and have had all the outcome measures) will be performed as the secondary analyses. Intention-to-treat analysis will be carried out by using multiple imputation [50], provided that patients have had a baseline MRI. This analysis only requires missing data to be missing at random to be valid. Differences within a treatment group between follow-up and baseline measures will be assessed using a paired samples *t*-test. The clinical efficacy measures, that is, changes in pain and function, will also be analysed using the normalised area under the curve for difference in scores from baseline to month 24. Differences between treatment groups will be assessed using independent samples *t*-tests, ANCOVA (for continuous variables) or chi-squared tests (for dichotomous variables). Multiple linear regression for continuous endpoints or logistic regression for binary endpoints could be carried out as supplementary analyses for additional adjustment for imbalanced baseline factors. Analyses of treatment efficacy will be done by censoring participants at the time of any protocol deviation and developing a model for the probability of deviation, followed by weighted analyses using only the uncensored participants where the weights are the inverse probability of censoring. This produces estimates of treatment effect as if there were full compliance with the protocol in this trial and is far preferable to per protocol analyses based on (unweighted) observed compliance [51]. Pre-specified analyses to identify subgroups which may respond better to treatment will be examined using stratified analyses; variables include radiographic OA and co-pathology present on MRI.

### Data integrity and management

All collected data are recorded using case report forms which will be processed centrally at the Clinical Informatics and Data Management Unit, Department of Epidemiology and Preventive Medicine, Monash University. The hard copies of the case report forms will be stored in a locked area at each study site with secured and restricted access. The electronic data will be stored in a password protected database with secured and restricted access. All data collected will be kept strictly confidential. Data transfer will be encrypted with all data de-identified.

### Withdrawal

If participants withdraw from the study before 2 years of follow-up, the reason and date will be recorded. If the participant withdraws after a minimum of 6 months of treatment, he/she will be requested to have a second knee MRI scan and complete the questionnaires.

### Monitoring

The principal investigators will monitor the conduct and progress of the project at each site. The trial coordinator will visit each study site to make sure that all trial procedures are compliant with the trial protocol. The principal investigators and the research team will have regular teleconferences to ensure efficient study execution and ongoing monitoring of the study progress, with summary documents circulated after each meeting. An independent Data and Safety Monitoring Board was convened, consisting of a clinical rheumatologist, a clinical cardiologist experienced in statin use, and a biostatistician, all with clinical trial experience. They will monitor adverse events. They will meet annually or more often if severe adverse events occur, and provide a written report to the principal investigators.

## Discussion

We proposed a multicentre, randomised, double-blind, placebo-controlled trial to determine whether atorvastatin has a disease modifying effect in symptomatic knee OA by slowing the structural progression of knee OA and improving knee symptoms. If atorvastatin is proven to be effective, it will offer a novel therapeutic approach to reducing the progression of knee OA.

Oral administration of statins is an established, safe and well-tolerated treatment for the prevention of cardiovascular events [23]. Extending the potential use of statins to patients with knee OA is plausible. There is *in vitro* and *in vivo* evidence that statins may reduce the progression of OA via a number of pathways including their effects on lipid metabolism and inflammation. Statins reduce the levels of C-reactive protein and the production of inflammatory cytokines including IL-6 and IL-1β, most likely through their inhibition of NF-kB activation in monocytes or endothelial cells exposed to inflammatory stimuli [52]. Statins inhibit IL-1β induced production of matrix metalloproteinases (MMPs), and stimulate bone morphogenetic protein 2, aggrecan, and synthesis of type II collagen and cartilage matrix proteoglycan by chondrocytes [53], which is protective against cartilage damage. Atorvastatin inhibited IL-1beta-induced glycosaminoglycan release, TNF-alpha, MMP-13, and superoxide anion formation, protecting cartilage degradation following IL-1beta-stimulated cartilage in an *in vitro* OA model [54]. In animal models, statins reduce inflammatory cell infiltration and matrix-degrading enzyme expression and inhibit pro-inflammatory cytokines, thus reducing articular cartilage degeneration and the severity and progression of OA or arthritis [55–58]. In an anterior cruciate ligament transaction induced rabbit OA model, intra-articular statin injections reduced the gross morphological and histological changes in articular cartilage [55]. In a mouse model resembling human lipoprotein metabolism, atorvastatin significantly suppressed OA development [59]. In a rabbit model of early experimental OA, intra-articular application of atorvastatin showed chondroprotective effects both macroscopically and histopathologically [60].

The previous human studies [25–30] (Table 1) are all of an observational nature and thus susceptible to selection bias, information bias and confounding, and have used insensitive tools to assess disease progression. Most of these studies examined general OA without stratifying by different joints, since emerging evidence suggests different pathogenic mechanisms of OA in knee and hip joints, with the knee affected more by metabolic factors than the hip [7]. A randomised controlled trial is required to determine whether statin use affects the structural progression of knee OA.

Although OA is a disease of the whole joint, progressive articular cartilage loss is the hallmark of disease progression. Radiographic joint space narrowing is the gold standard to assess disease progression over time and has been used as the primary endpoint to examine the effect of disease modifying OA drugs in clinical trials. However, radiographic joint space narrowing provides a crude, insensitive method to assess disease progression [36]. MRI allows non-invasive direct visualisation of all joint components and direct measurement of articular cartilage, representing a sensitive method to assess OA progression. Cartilage volume has been validated by comparison with anatomical specimens [61]. It is a clinically useful measure which is inversely correlated with radiological grade of OA [41]. Cartilage volume loss is clinically significant, as it predicts important patient outcomes of pain [62] and risk of joint replacement [48]. Cartilage defects have been validated against surgically confirmed cartilage lesions and arthroscopy [63]. They are associated with knee pain [12], predict cartilage loss independent of cartilage volume [64], and predict joint replacement [49], thus providing a further assessment of cartilage health. Being the most common subchondral bone abnormality described in OA, BMLs are associated with knee pain [65], predict disease progression [66], cartilage volume loss [13] and joint replacement [14]. Thus, these MRI-assessed knee structural changes have been chosen as the primary and secondary outcome measures in this study.

In summary, knee OA is the most common form of chronic joint disorder but has no proven pharmacological treatment that reduces structural disease progression. There is evidence that both inflammation and lipid metabolism contribute to the progression of OA. The anti-inflammatory and lipid lowering effects of stains make this class of drug a potential treatment approach to slowing the progression of OA. This randomised controlled trial will provide high-quality evidence to address whether atorvastatin has a novel disease modifying effect in OA by delaying the structural and symptomatic progression of knee OA. If demonstrated to be effective, atorvastatin could be used as a novel disease modifying agent to delay the onset of end-stage OA and the need for primary joint replacement for OA and possibly revision surgery. This has major public health importance, as it would produce substantial cost savings by delaying and possibly reducing the need for joint replacement surgery, and provide marked improvements in quality of life for people with knee OA.

### Trial status

Upon submission, the OAKS study is in the process of patient recruitment.

## Abbreviations

ACR: American College of Rheumatology
BML: bone marrow lesion
HDL: high-density lipoprotein
HMG-CoA: hydroxy-methyl-glutaryl coenzyme A
IL: interleukin
IPAQ: International Physical Activity Questionnaire
LDL: low-density lipoprotein
MMP: matrix metalloproteinase
MRI: magnetic resonance imaging
OA: osteoarthritis
OAKS: Osteoarthritis of the Knee Statin
TNF: tumour necrosis factor
VAS: visual analogue scale
WOMAC: Western Ontario and McMaster Universities Osteoarthritis Index
